# Measuring social phobia symptoms in a community sample of adolescents: An examination of the psychometric properties of the SPAI-23

**DOI:** 10.3389/fpsyg.2022.1002221

**Published:** 2022-12-21

**Authors:** Markos Apostolakis, Marios Theodorou, Klavdia Neophytou, Georgia Panayiotou

**Affiliations:** ^1^Department of Psychology, University of Cyprus, Nicosia, Cyprus; ^2^Center for Applied Neuroscience, University of Cyprus, Nicosia, Cyprus

**Keywords:** social phobia, social anxiety, SPAI, assessment, confirmatory factor analysis, exploratory factor analysis, adolescence

## Abstract

A number of studies to date examine dimensions of social phobia and anxiety in adolescents. A variety of tools has been developed, along with their abbreviated versions, that are used to assess Social Anxiety (SA) but little research has been devoted to the types of fears they each assess. Due to differences in the content of the multitude of instruments, different aspects of SA are addressed and this leads to confusion when the relationship between SA and other constructs is being investigated. The aim of the present study was to examine the psychometric properties of the abbreviated Social Phobia and Anxiety Inventory SPAI-23 in Greek-Cypriot community adolescents and describe dimensions of social fears at that age. Seven hundred twenty-one adolescent students from Cyprus, (Mean Age: 15.5, Range: 13–19, SD: 1.12, 64% female) participated in the study. Participants completed, among others, an abbreviated version of the Social Phobia and Anxiety Inventory (SPAI-23). Exploratory Factor Analysis on the SPAI-23 revealed a quite similar structure to the original questionnaire (SPAI). Three Social Phobia factors, describing distinct socially fearful situations, were identified (Performance, Interaction, and Presence in a social context) and one Agoraphobia factor after the evaluation of alternative solutions. Findings were verified by means of Confirmatory Factor Analysis, testing alternative models. Overall, findings were in line with recent evidence on youth samples, and contribute to significant insights towards more sophisticated and personalized assessments.

## Introduction

Social Anxiety Disorder (SAD), characterized by discomfort during actual or anticipated social situations, is highly prevalent in childhood and adolescence, and significantly increases the risk for socioemotional maladjustment later in life (Degnan et al., 2010; Fox and Pine, 2012). SAD typically develops during late childhood - early adolescence (Beesdo et al., 2007; Kessler et al., 2007; Burstein et al., 2011; Leigh and Clark, 2018) and, if left untreated, persists in adulthood and negatively impacts quality of life (Van Ameringen et al., 2003; Ryan and Warner, 2012). Even subclinical levels of social anxiety (SA), that do not meet criteria for SAD diagnosis, can be quite distressing and cause difficulties in multiple life domains of adolescents, including academic (Ranta et al., 2009), forming friendships and peer and romantic relationships. Socially anxious youth tend to form relationships of lower quality (La Greca and Harrison, 2005; Hebert et al., 2013) and are at increased risk of peer victimization (Ranta et al., 2009; Acquah et al., 2016).

Social anxiety, whether clinical or subclinical, can be manifested in a wide range of situations. Socially anxious individuals may feel anxiety in only a few social situations or most/all social situations (Hofmann et al., 2004; Vriends et al., 2007), something that may reflect a continuum of severity (Bögels et al., 2010). Whether this diversity in the contexts where symptoms are manifested and the types of symptoms one predominantly displays represent SA subtypes or diverse phenomenologies of the same disorder, has been debated in the literature, but the consensus, as shown in DSM-5 is that these are not real subtypes due to similar etiologies and response to treatment and the fact that the majority of individuals with SA are anxious in multiple situations (Heimberg et al., 1993). Nevertheless, knowing one’s unique profile of specific social fears is important in designing personalized treatments for people with SAD, and helping those with subclinical SA develop strategies to cope with everyday challenges.

In the case of performance fears, where the individual presents with anxiety in performance situations only, e.g., speaking in front of an audience, knowing that anxiety is circumscribed to such settings is critical for diagnosis and case formulation. Fear of performance constitutes a unique diagnostic specifier in DSM-5 for SAD, and individuals with this form of the disorder may have unique characteristics. According to Hofmann et al. (2004) and Hook and Valentiner (2002) people with performance anxiety are qualitatively distinct in that they are more similar to people with specific phobias in terms of heredity, psychophysiological response to feared situations, onset and predisposing risk factors.

In fact, beyond the well-established Performance Only specifier, included in DSM-5, multiple studies suggest the presence of distinct domains of anxiety-provoking social situations, based typically on factor analyses of responses to social anxiety psychometric tools, which typically yield three to five factors. These include “fear of interaction,” e.g., dating, “fear of observation” e.g. being watched when eating in front of others (Cox et al., 2008; Bögels et al., 2010) and additional factors pertaining to the predominance of physical and somatic symptoms vs. avoidance (Cederlund and Öst, 2013; Panayiotou et al., 2017). Research has not yet reached consensus regarding the number or content of SA subdimensions and existing research has focused primarily on adult populations (Dalrymple and D’Avanzato, 2013).

Factor analytic studies of SA psychometric tools typically yield between one and five dimensions, reflecting different situations where symptoms appear, as well as types of symptoms experienced. Knappe et al. (2011) assessed 3,201 youth between 14 and 24 years old using a computer-assisted version of the Munich-Composite International Diagnostic Interview (DIA-X/M-CIDI), conducted an Exploratory Factor Analysis (EFA) and identified one general factor. Mörtberg and Jansson Fröjmark (2019) run a study using the Social Phobia Inventory (SPIN) in a young adult sample, and reported two factors; fear and avoidance of social interaction and fear and avoidance of criticism. A two-factor model was also reported by Ouyang et al. (2020), who examined the factor structure of the Social Interaction Anxiety Scale (SIAS) and Social Phobia Scale (SPS) in a young adult sample, similar to Zsido et al. (2021) who tested the same scales in adults and adolescents. Panayiotou et al. (2017) administered the Social Phobia and Anxiety Inventory (SPAI) (Turner et al., 1989) to a sample of young adults to evaluate its psychometric properties and then compared it, using CFA, with several models of previous studies and a preliminary EFA. The results revealed four correlated Social Phobia factors, an agoraphobia factor and four situation factors defining the context in which symptoms are expressed. In this study, of particular importance is that the items of the best fitting model were allowed to load on two factors, a subdimension of social fears factor (e.g., Social Interaction, Focus of Attention) and a situation factor (e.g., Strangers, Authority Figures). This suggests that a bifactor structure may be better at explaining social fear subdimensions in SPAI. Lastly, Schry et al. (2012) used the SPAI-23 with an adult sample and identified two different models both of which fit the data well: A two-factor model, where the factors were social anxiety and agoraphobia, and a three-factor model, consisting of factors representing social anxiety, public speaking anxiety, and agoraphobia. However, it is not known how this questionnaire performs in an adolescent population and whether the same factors would appear.

Research with youth, adolescents and children has also yielded different numbers of factors on a variety of screening tools that adequately detect social anxiety (García-López et al., 2015) and there is some consistency in the dimensions describing anxiety in specific situations, interactions or other symptoms. Cederlund and Öst (2013) administered the Social Phobia and Anxiety Inventory for Children (SPAI-C) to 59 youth between 8 and 14 years old fulfilling the criteria for SAD (based on DSM-IV), and identified three latent factors using EFA: (1) social interactions, (2) public performance situations, (3) physical and cognitive symptoms related to social anxiety. Five social anxiety factors were identified by Aune et al. (2008), who administered SPAI-C in a sample of 2,148 students (11 to 14 years old) and conducted initially an EFA and 1 year later confirmatory factor analysis (CFA). These five factors were labelled as: (1) Assertiveness, (2) Public Performance, (3) Physical/Cognitive Symptoms, (4) Social Encounter and (5) Avoidance. In addition to EFA approaches, Piqueras et al. (2008), tested 971 adolescents, from which 795 fulfilled the criteria for SAD, between the ages 14 and 18 using the Anxiety Disorder Interview Schedule for DSM-IV, Lifetime Version (ADIS-IV-L), conducted Principal Component Analysis (PCA) which identified two factors: interaction and performance. They also conducted a cluster analysis of the participants which grouped them into four subgroups; the 1st group with specific social phobia, the 2nd with mild generalized social phobia, the 3rd with moderate generalized social phobia and the last group with severe generalized social phobia. Thus, the two subtypes of SAD that were suggested were “specific social phobia” and “generalized social phobia.”

As the studies above demonstrate, social anxiety and its subdimensions has been extensively investigated with several psychometric instruments, among which the well-established Social Phobia and Anxiety Inventory (SPAI) (Turner et al., 1989), which has been proven to be a very reliable tool in the assessment of SA in general and clinical populations (Beidel et al., 1989; Herbert et al., 1991; Peters, 2000; Bunnell et al., 2013). It consists of a Social Phobia (SP) subscale that contains 32 items and an Agoraphobia (AG) subscale that contains 13 items and assesses anxiety in a wide range of situations and settings. It has been translated and utilized in several countries, e.g., the Netherlands (Bögels and Reith, 1999), Spain (García-López et al., 2001), Cyprus (Panayiotou et al., 2017) and others, in which its psychometric characteristics in clinical and community samples have been demonstrated.

Several abbreviated versions have been created to reduce administration time and all have turned out to be highly reliable, with psychometric properties comparable to those of the original version. SPAI-18 (de Vente et al., 2014) contains 18 items all stemming from the Social Phobia subscale and assesses all five aspects of social anxiety included in the original SPAI (social situations, center of attention, avoidance, cognitive symptoms and somatic reactions). Reliability for community individuals was *α* = 0.93 and patients *α* = 0.91, and it correlated highly with the social phobia subscale of SPAI, *r* = 0.98. SPAI-B (García-López et al., 2008) contains 16 items assessing cognitive behavioral and somatic symptoms of the social phobia subscale. SPAI-B correlated highly with SPAI (*r* = 0.76) and was highly reliable in a community sample of adolescents (*α* = 0.92). SPAI-C (Beidel et al., 1995) was specifically designed to be administered in children and it contains 26 items along with sub-items from the social phobia subscale of SPAI. It assesses physical, cognitive and behavioral characteristics of SA and reliability was high (*α* = 0.95).

In this study, SPAI-23 (Roberson-Nay et al., 2007) was used, which has 23 items, 16 measuring Social Phobia and 7 measuring Agoraphobia. It was developed using item response theory (IRT), instead of the commonly used classical test theory (CTT), which allowed the authors to assess responses to each item of the scale and the performance of the scale overall and, thus, select the items from the SP and AG subscales that best measure these constructs. A major advantage compared to other abbreviated versions is that it contains both SP and AG items and item selection was based on methodological rigor. Additionally, both SP and AG subscales correlate highly with those of SPAI (*r* = 0.97 and *r* = 0.90 respectively). Demonstrating that this tool is psychometrically solid and yields similar sub-factors obtained from other instruments, when used with adolescents, can increase the usability of this well-constructed instrument for young populations. Furthermore, assessing its factor structure will contribute new evidence with regards to the dominant social fears and social anxiety symptoms experienced by adolescent populations.

Social anxiety has also been found to correlate with a number of vulnerability factors and temperament characteristics in both adults and youths (e.g., Mick and Telch, 1998; Panayiotou et al., 2014), which may have meaningful associations with specific social anxiety dimensions. Associations between observed SA dimensions and these well-established correlates of SA can add credibility to the observed factor structure of the SPAI and its abbreviations and suggest hypotheses regarding the mechanisms that may drive each symptom category. More specifically, temperamental traits, specifically Behavioral Inhibition, predicts SA directly and interaction fears are particularly related to it (Degnan et al., 2010; Knappe et al., 2011; Panayiotou et al., 2014). Anxiety Sensitivity, another temperamental predictor of SA, maintains symptoms of clinical levels of SA *via* a tendency to avoid undesirable experiences (Experiential Avoidance), which mediates its predictive role (Orsillo et al., 1994; Panayiotou et al., 2014; Papachristou et al., 2018). Additional vulnerability factors, including Psychological Inflexibility are positively associated with SA or agoraphobia in samples of adults, children, and adolescents (Muris, 2002; McLaughlin et al., 2007; Levin et al., 2014; Tillfors et al., 2015; Simon and Verboon, 2016; Papachristou et al., 2018). Experiential avoidance and psychological inflexibility are considered malleable factors that can be addressed through psychological interventions, and therefore are particularly useful to identify as predictors of SA, as addressing these may lower the risk conferred by temperamental characteristics. Lastly, using psychophysiological indices, Panayiotou et al. (2017) suggested that fear of public speaking may be more akin to a specific phobia, while the more generalized SAD subtype may reflect generalized distress rather than fear. This leads to the prediction that these different fear categories suggest alternative maintenance mechanisms that can operate as putative vulnerability factors for developing SAD later in life.

The present study aims to extend research on social anxiety dimensions and social fear clusters, in a non-clinical community sample of adolescents, by examining for that purpose the factor structure of a commonly used instrument, the SPAI-23, and evaluating the relationship between vulnerability factors and temperamental characteristics and SPAI-23 factors. It was expected that the extracted factors of the abbreviated version of the Social Phobia and Anxiety Inventory (SPAI-23) would reflect the structure of the full version (SPAI) and confirm it by means of confirmatory factor analysis (CFA) as well as bifactor CFA. Given that SPAI-23 does not include specific situations for each symptom, specified bifactor models would not distinguish between social anxiety dimensions and situations, rather a general Social Phobia factor because the primary aim of an abbreviated questionnaire, apart from reducing administration time, is to maintain its capacity to assess overall symptomatology and additional factors would include domains of Social Anxiety. Specifying bifactor models would allow us to define a general factor representing the main construct of interest (SP) and specific factors explaining variance other than that accounted for by the general factor (Reise et al., 2010). Taken that SPAI is a widely used questionnaire, the factor structure should resemble the most commonly reported SA dimensions, and, therefore, it was expected that results would confirm the validity and reliability of the Greek translation and its capacity to detect the most common SA dimensions, proving that it is a valuable tool for quick administration. Lastly, it was expected that behavioral inhibition, anxiety sensitivity and psychological inflexibility would positively correlate with SA dimensions but given the unclear previous results, the relationships will be explored.

## Materials and methods

### Participants

Seven-hundred twenty one (433 female) Greek-Cypriot high-school students from five districts of the Republic of Cyprus participated in the study. Participants’ age was between 13 and 19 (mean age = 15.5, SD = 1.12). A stratified random sampling approach was used to select a representative sample of secondary schools (based on geographic area). Schools were selected from the rosters of the Ministry of Education. Then specific grades were selected from each school randomly. All students from the selected grades were invited to participate in the study. Only students whose parents gave written consent, participated in the study. The study received approval from the Cyprus National Bioethics Committee and from the Ministry of Education of the Republic of Cyprus. Data on the demographic characteristics of the sample show a similar distribution of subjects in rural (44.7%) and urban areas (54.3), most lived with both their parents at the time of testing (84.7%), fewer lived with one parent (14.6%) and 0.4% reported “other.” The educational level of mother and father had a different pattern, 29% of mothers had completed secondary education, 19.3% technical education and 17.8% higher education whereas 41% of fathers completed secondary education, 4.6% completed technical education and 20% higher education. Additionally, most subjects had between one and three more siblings (85.7% cumulative).

### Measures

#### Demographics

A number of items assessed demographic characteristics such as area of residence (rural or urban), household members and educational level of parents.

#### SPAI-23

SPAI-23 (Roberson-Nay et al., 2007) measures symptoms of Social Anxiety. It contains 23 items and includes two subscales; agoraphobia (7 items) and Social Phobia (16 items). The items are rated on a 5-point Likert scale and range from 0 to 4 (never to always). It is an abbreviated version of the SPAI questionnaire (Turner et al., 1989) which contains 45 items and assesses cognitive and somatic symptoms and behaviors in a wide range of situations that have the potential to elicit SA. Roberson-Nay et al. (2007), who created the abbreviated version based on data collected from young adults, reported strong factor loadings for all items, high correlations between the subscales of SPAI-23 and the original SPAI, it is comparable with other social anxiety measures similar to the original SPAI and, also, it adheres to a normal distribution better that the original version. The reduced number of items did not result in significant reduction of reliability, which was .95 for the Social Phobia subscale and.85 for the Agoraphobia subscale, and validity of the test scores (Roberson-Nay et al., 2007). Similarly, Schry et al. (2012) report strong psychometric properties; results from four studies with different populations, showed reliability >0.90 in the Social Phobia subscale and > 0.80 for the agoraphobia subscale. An exploratory factor analysis resulted in a two-factor model, where the factors were social anxiety and agoraphobia, and a three-factor model, in which the factors were social anxiety, public speaking and agoraphobia, and both models fit the data well (Schry et al., 2012). However, it is not known how this questionnaire performs with an adolescent population as this is one of the first studies evaluating SPAI-23 psychometric properties in youth.

#### BIS/BAS

The Behavioral Inhibition System/Behavioral Activation System scale (Carver and White, 1994) assesses two basic motivational systems underlying appetitive and aversive behavior. It contains 20 items on a four-point Likert-type questions, ranging from “totally disagree” to “totally agree.” It consists of four subscales, one BIS subscale (7 items) and three BAS subscales (13 items), i.e., the Punishment Sensitivity subscale (BIS), the Drive subscale (BAS), the Fun Seeking subscale (BAS) and the Reward responsiveness subscale (BAS). Its reliability has been deemed acceptable (see Table 1 for reliability in the current sample), e.g., Carver and White (1994) found the reliability of the subscales in an adult population to be between.73 and 0.76 for the BIS, Reward Responsiveness and Drive subscales, and 0.66 for the Fun Seeking subscale. A recent study with adolescents as the sample (Vandeweghe et al., 2016) report similar αs, 0.74 for the BIS subscale and.70 for all BAS subscales. The scale has been validated in the Greek Language in an adolescent sample by Kokkinos and Voulgaridou (2017) and demonstrated good psychometric properties with *α* = 0.75 for the BIS subscale and *α* = 0.79 for the BAS subscale.

**Table 1:** Descriptives and Cronbach’s alphas for each scale with the current sample.

	SPAI-23	AFQ-8	CASI	BIS/BAS
Mean	0.90	1.12	0.67	1.32/1.82
Median	0.82	1	0.67	1.28/1.84
SD	0.53	0.81	0.38	0.55/0.65
Skewness	0.70	0.91	0.40	−0.19/−0.65
Kurtosis	0.34	0.56	−0.17	−0.27/0.09
Cronbach’s *α*	0.92	0.84	0.88	0.67/0.90

#### AFQ-Y8

The Avoidance and Fusion Questionnaire-Youth 8-item scale (AFQ-Y8) (Greco et al., 2008) is measuring psychological inflexibility (PI) in children and adolescents. It contains 8 items and responses are given on five-point Likert-type questions, ranging from 0 “not at all true” to 4 “totally true.” It is an abbreviated version of the 17-item AFQ-Y (Greco et al., 2008) which assess PI engendered by cognitive fusion (*CF*) and experiential avoidance. A study investigating the psychometric properties of the Greek translation of the AFQ-Y8 reported excellent Cronbach’s alpha level, 0.87, (see Table 1 for reliability in the current sample; Christodoulou et al., 2018) similarly to the original version (Greco et al., 2008).

#### CASI

The Childhood Anxiety Sensitivity Index (CASI) (Silverman et al., 1991) measures anxiety sensitivity in children. It contains 18 items and responses are given in three-point Likert-type questions with responses ranging from 0 “not at all” to 2 “a lot.” Psychometric evaluation of the scale shows adequate and acceptable internal consistency in samples of children and adolescents of Dutch and Catalan origin (van Widenfelt et al., 2002; Fullana et al., 2003) as well as in the current sample (Table 1). Adaptation in the Greek language has been performed for the current study by means of front and back translation (see Papachristou et al. (2018), for a detailed description).

#### Procedure

After gaining school permission, the research team initially visited each school to provide students with invitations and informed consent forms to take home. The students who provided a written consent from both their self and parents, were eligible to participate in the study. Then, Participants completed a self-report paper-and-pencil questionnaire package, in a classroom format, during school-hours. The questionnaire completion took approximately 45 min. During the data collection a research assistant and a school teacher were present in order to answer questions when necessary and ensure confidentiality and independent responding.

#### Statistical analyses

All data were entered in SPSS (IBM Corp. Released, 2017. IBM SPSS Statistics for Windows, version 25.0. Armonk, NY: IBM Corp.) and were initially screened for missing values. No cases were deleted as missing values per item did not exceed 1.1%. Next, data were assessed for multivariate outliers based on Mahalanobis distance (Tabachnick and Fidell, 2013) and 41 cases were excluded, resulting in a sample of 680 subjects. Internal reliability indices for measures used in the current study were calculated (Table 1).

To our knowledge, no other study has investigated the factor structure of the SPAI-23 in adolescents and, therefore, exploratory factor analysis was selected as the first step of our analyses and then a confirmatory factor analysis was carried out to test current findings and previous studies. The analytic procedure was based on previous findings, i.e., factor structure of SPAI-23 in adults, and the conceptual framework around Social Anxiety, i.e., suggested SA subtypes in the literature. More specifically, an exploratory factor analysis, using principal axis factoring (PAF) and oblique rotation, because the factors were expected to be correlated, were applied and models with two to four factors were explored for best fit on the data. For the sake of comparability with the two previous latent factor evaluations of the SPAI-23 in adults, the same analytic adjustments were made, that is, it was required that items should load >0.30 on their primary factor, items were required to have <0.30 cross-loadings on secondary factors and, lastly, in order for a factor to be accepted it should have included more than two items with a loading >0.30 (Roberson-Nay et al., 2007; Schry et al., 2012).

## Results

### Exploratory factor analyses

A series of exploratory factor analyses were carried out to examine different models and identify the best fitting model to the data. At first, restricted EFAs to two factors and three factors, based on previous studies (Roberson-Nay et al., 2007; Schry et al., 2012) and analysis strategies were performed, and then an unrestricted EFA. In all EFAs, principal axis factoring (PAF) and an oblique rotation (Oblimin) were used. Maximum likelihood estimator was also attempted as in Schry et al. (2012) but results were not meaningful and were rejected.

The two-factor constrained EFA (Table 2) was carried out because SPAI-23 contains an SP subscale and an AG subscale, which were expected to be shown. The two factors that were extracted both had an Eigenvalue over 1, the factor loadings were 0.3 or more and explained 41.6% of the variance. Factor one contained all items of the SP scale and factor two all items of the AG subscale, as expected. Three items (8, 13, and 16) had cross-loadings of 0.30 to 0.35. No item failed to load on a factor. A three-factor constrained EFA (Table 3) was carried out to replicate Schry et al. (2012). The extracted factors had eigenvalues above 1 explaining 38.5% of the variance, all items loaded on at least one factor and factor loadings were 0.3 or more, two items (items 20 and 19) loaded in two factors. Factor 1 termed “Social Anxiety” (SA) contained 15 items, factor 2, “Agoraphobia” contained all items of the agoraphobia subscale and the third factor contained items related to public performance and, thus, labelled “Performance” (Perf) factor. The unrestricted EFA resulted in four factors (Table 4) with an Eigenvalue over 1 and explained 48.3% of the variance. The factor loadings were ≥ 0.33 and no item failed to load on a factor. Factor one contained eight items and was labeled “Social presence” (Sp), factor two contained the seven items of the AG subscale, thus it was labelled “Agoraphobia,” factor three had three items and was labelled “Performance” (Perf) and the fourth factor contained five items and was labelled “Interaction” (Int). One item (item 3) cross-loaded in the factors one and three. Given that this item contains two interconnected statements that semantically fit in both factors it is reasonable to have this cross-loading. This solution was considered as best fitting to the data but, also, reflects the general direction in the literature regarding social fear subtypes. Lastly, Cronbach’s alphas for all extracted factors of all solutions and correlations were calculated (Table 5).

**Table 2:** Results of a two factor solution from a forced EFA using PAF and oblique rotation.

SPAI-23 item	Factors and factor loadings	
	Social Phobia	Agoraphobia
2	0.77	
3	0.76	
1	0.74	
5	0.71	
6	0.65	
7	0.65	
4	0.64	
14	0.53	
9	0.52	
15	0.51	
13	0.44	0.34
10	0.44	
12	0.41	
16	0.41	0.35
11	0.32	
19		0.66
22		0.65
20		0.60
18		0.54
21		0.50
23		0.49
17		0.49
8	0.30	0.39

Factor loadings > 0.30 are listed.

**Table 3:** Results of a three factor solution from a forced EFA using PAF and oblique rotation.

SPAI-23 items	Factors and factor loadings		
	Social anxiety	Agoraphobia	Performance
13	0.82		
7	0.70		
14	0.67		
1	0.66		
2	0.64		
6	0.64		
12	0.63		
8	0.62		
16	0.60		
10	0.56		
15	0.55		
9	0.52		
11	0.47		
20	0.42	0.36	
22		0.63	
18		0.59	
21		0.56	
17		0.52	
19	0.39	0.42	
23		0.40	
5			0.70
4			0.60
3			0.56

Factor loadings > 0.30 are listed.

**Table 4:** Results of a four factor solution from unrestricted EFA using principal axis factoring (PAF) and oblique rotation.

SPAI-23 item	Factors and factor loadings			
	Social Presence	Agoraphobia	Performance	Interaction
2	0.77			
7	0.73			
1	0.64			
6	0.59			
16	0.41			
8	0.35			
9	0.33			
15	0.32			
22		0.65		
18		0.60		
21		0.56		
17		0.55		
19		0.52		
20		0.47		
23		0.46		
5			0.65	
4			0.60	
3	0.40		0.47	
12				0.74
10				0.67
13				0.66
11				0.65
14				0.55

Factor loadings > 0.30 are listed.

**Table 5:** Correlations between factors for each solution and Cronbach’s alphas for each extracted factor.

Solution	1		2			3			
Factor	SP	AG	SA	AG	Perf	Sp	AG	Perf	Int
1									
2	0.55								
1									
2			0.58						
3			0.46	0.14					
1									
2						0.47			
3						0.33	0.16		
4						0.63	0.58	0.33	1
Cronbach’s α	0.91	0.80	0.91	0.75	0.78	0.86	0.78	0.78	0.82

### Confirmatory factor analyses

A series of CFA models (Table 6) using AMOS 27.0 were evaluated to test previous studies, theory and current study’s EFAs. To evaluate the models the following indices were utilized: χ^2^ and df to assess overall fit, Root Mean Square Error of Approximation (RMSEA) in which a value <0.08 and preferably <0.05 show good fit (Hu and Bentler, 1999), Comparative Fit Index (CFI) where a value >0.90 indicates good fit, Akaike’s (1987) Information Criterion (AIC), Consistent AIC (CAIC) and Bayes Information Criterion (BIC) which assess model parsimony and smaller values indicate better fit.

**Table 6:** Confirmatory factor analyses results.

Model	Fit indices						
	*χ* ^2^	Df	CFI	RMSEA	AIC	CAIC	BIC
1	1586.829**	229	0.80	0.093	1680.82	1940.36	1893.36
2	1343.686**	227	0.83	0.085	1441.68	1712.26	1663.26
3	1129.133**	224	0.86	0.077	1233.13	1520.28	1468.28
4	755.167**	201	0.91	0.064	905.16	1319.32	1244.32
5	706.903**	192	0.92	0.063	874.90	1338.75	1254.75

**p < 0.001.

Model 1 was a two-factor model consisting of an SP factor and an AG factor. The purpose of this model was to test whether this shortened version of the questionnaire that does not include quadruple questions as the full version would replicate previous studies and that items would load in their respective factor. Items 1–16 loaded on a social phobia factor and items 17–23 on an agoraphobia factor. Fit indices were not acceptable, CFI was below recommended standards and RMSEA was higher than recommended standards. It is, therefore, assumed that these results replicate previous findings of inadequacy of a two-factor solution (SP and AG).

Models 2 and 3 evaluated a distinction of the SP items into more specific “situation” factors. In model 2, two factors were specified for the 16 SP items, distinguishing performance given in front of an audience (“fear of performance”) with 3 items and generic presence in a social context (“fear of presence in a social context”) with 13 items. An AG factor was specified for the 7 AG items. Fit indices were not acceptable despite an improvement compared to model 1. Model 3 included a further division of the SP items and 3 factors were specified, “fear of performance,” “fear of interaction” and “fear of presence in a social situation.” This model proved to be more parsimonious, based on information criteria, than the previous two and had acceptable fit indices apart from CFI which did not exceed 0.90.

Following this, bifactor models were specified to test the idea that SA severity falls along a continuum that is influenced by a number of feared situations and severity in those fears (Bögels et al., 2010). As a result, model 4 included the factors of model 3 and in addition, a general SP factor containing all 23 items. Model fit was acceptable but loadings on the “fear of presence in a social situation” were not significant and for this reason an additional model was specified, which separated the items of this factor in more coherent, thematically, categories. Model 5 had a generic SP factor including all items of the scale, 5 SP factors (“fear of small groups,” “fear of performance,” “fear of large groups,” “fear of interaction” and “anxious thoughts”) and 1 AG factor. Fit indices surpassed acceptable standards and were superior to the other models, this model was more parsimonious than all previous models and factor loadings were significant.

### Correlates of social anxiety subfactors

Pearson correlation coefficients were computed (Table 7) to assess the relationship between SPAI-23 and subfactors of it with measures of psychological inflexibility, anxiety sensitivity and the behavioral inhibition/behavioral activation system. All subfactors correlated significantly with the AFQ, CASI and BIS with *r* fluctuating between 0.29 and 0.49 whereas correlations with the BAS scale were much lower, between 0.8 and 0.17, and also non-significant, i.e., with fear of interaction. At the same time, when examining correlation between SPAI-23 and these scales, all relationships are positive and significant but, again, BAS has the lowest correlation coefficient (*r* = 0.15, *p* < 0.01). It was, additionally, tested how the Social Phobia factor as a whole would relate to AFQ, CASI, BIS and BAS. Correlations were positive and moderate, between.15 and.49 (*p* < 0.01) with BAS having the lowest value (*r* = 0.15).

**Table 7:** Correlations between personality characteristics and SPAI-23 and subfactors.

	Cronbach’s α	AFQ	CASI	BIS	BAS
Full SPAI-23	0.92	0.43**	0.42**	0.33**	0.15**
Social Phobia	0.92	0.49**	0.53**	0.38**	0.15**
Fear of Performance	0.78	0.42**	0.43**	0.36**	0.17**
Fear of Interaction	0.83	0.37**	0.41**	0.25**	0.05
Fear of presence in a social context	0.87	0.47**	0.49**	0.35**	0.15**
Agoraphobia	0.78	0.37**	0.47**	0.29**	0.08*

AFQ, Avoidance and Fusion Questionnaire; CASI, Child Anxiety Sensitivity Index; BIS, Behavioral Inhibition Scale; BAS, Behavioral Activation Scale; SP, Social Phobia; AG, Agoraphobia; *p < 0.05, **p < 0.01

## Discussion

This research was conducted in response to the prevalence and persistence of SA, from a young age through adulthood, which emphasizes the need for early and valid diagnosis to prevent later dysfunction. There is a need for cost-and time-effective screening tools to assist practitioners in personalizing treatment approach and to assist researchers in further understanding SA through extensive screening of the general population, as well as individual assessment to identify personalized difficulties and needs. In this study, the factor structure of the Greek version of SPAI-23 was examined and this is the first study, to our knowledge, in which it was administered to a community sample of adolescents. The aim of this study was manifold: to validate the SPAI-23 in the Greek language in a community sample of adolescents, to identify dimensions of social fears in adolescents that explain the heterogeneity of difficulties observed in SA, to compare them with dimensions of social fears observed in adults and to provide insights on the developmental trajectory of SA. An additional goal was to investigate risk factors that represent correlates of social fears and may contribute in the development and maintenance of SA.

A series of EFA were carried out to replicate previous findings from adult studies and explore the factor structure of SPAI-23 in a Greek speaking adolescent community sample. The first solution was in accordance with the structure of SPAI-23 (Roberson-Nay et al., 2007), meaning that all items of the Social Phobia subscale loaded in one factor and all items of the Agoraphobia subscale loaded in a second factor. Next, it was decided to further investigate potential subgroups of items that measure distinct clusters of SA behaviors, which emerged in a previous study (Schry et al., 2012) and the results were replicated for the most part. The “agoraphobia” factor emerged identical and a “social anxiety” factor that contained the same items apart from two was also replicated; a third factor referring to performance (in front of an audience or group of people) contained the same items as the “public speaking” factor found by Schry et al., apart from one. The unrestricted EFA revealed a factor structure resembling some of the most commonly identified social fears but due to the reduced number of items it does not include specific situations that are reported in more severe cases of SA (Crome and Baillie, 2014), suggesting that SPAI-23 is a tool suitable for large scale screenings.

Furthermore, a series of CFAs were conducted to test the EFA results as well as replicate previous findings and theory. Model fit of Model 1 did not meet acceptable standards, and, thus, does not support a two-main-factor structure and suggests instead that additional factors may improve fit, which is in accordance with Panayiotou et al. (2017). Models 2 and 3 showed an improvement without reaching acceptable levels but confirmed the idea that additional factors may better explain the data. As a next step, bifactor models were specified because severity of SA correlates with the number and range of social fears (Bögels et al., 2010; Skocic et al., 2015). As a result, two models with a general Social Phobia factor in which all items loaded and additional subfactors were specified; Model 4 with four subfactors and Model 5 with six subfactors. The decision for a larger number of factors was based on Panayiotou et al. (2017), who defined models with multiple situation factors. Model fit was similar and surpassed acceptable standards in both occasions, indicating that variance not explained by more specific subfactors is accounted for by a general factor. These findings partially support Osman et al. (1995) that all SP items load a single factor but, also, the contrasting findings of Olivares et al. (1999) who rejected a single factor solution and proposed rather a multifactorial structure. In addition, the scale as a whole and the individual factors had very good internal consistency suggesting that SPAI-23 has sufficient reliability to be administered for research and screening purposes to adolescents. In all models the Agoraphobia factor was retained as is, because one of the primary aims of the study was to examine the psychometric properties of the questionnaire, and, thus, it was necessary to keep intact all parts and test all items.

Expression of SA varies depending on feared contexts (Panayiotou et al., 2017). Whether these map onto subtypes of SAD (Kodal et al., 2017), and what their characteristics are has been a persistent debate in the clinical literature. The debate resulted in the proposal for a general performance-only specifier (Bögels et al., 2010; American Psychiatric Association, 2013), rather than the support of different subtypes. Expression of SA varies also depending on symptom type and severity. It has been reported that symptoms such as taking exams or being interviewed indicate mild SA whereas fear of more specific interactions such as arguing with unfamiliar people indicate more severe cases (Crome and Baillie, 2014). Crome and Baillie (2014) suggest that fear of activities that are more likely to occur in everyday life (such as eating in public) compared to rarer activities (such as speaking in front of an audience) cause more difficulties in a person’s life and also that the more severe SA is, the more irrational the fears are. It is therefore, debatable, whether an abbreviated questionnaire can capture accurately more severe SA cases when it does not contain items with such specificity. Nevertheless, the observed variation in social fears and symptoms (type and severity) may lead to unique profiles, that need to be considered when designing more personalized interventions. Lastly, not only patients but also individuals who do not surpass diagnostic thresholds and are experiencing debilitating distress, impairment and, possibly, comorbidity (Fehm et al., 2008) may present distinct profiles based on their SA symptoms, which is also supported by the conception of SA as a dimensional construct (Crome et al., 2010).

Pairwise comparisons between SPAI-23 factors and measures of personality and temperamental characteristics indicate positive relationships of low to moderate strength, with the only exception being Behavioral Activation with which correlations were too low, though significant, with most factors. Pairwise correlations do not allow for interpretations regarding mechanisms of development and maintenance of social fears but suggest instead that further research is required to examine these relationships. In particular, the connections between SA factors and temperament need to be further addressed because this gap in the literature is even more pronounced in community youth studies, whereas the relationship between temperament and SAD is thoroughly being investigated. Here, the results were as expected, based on the extensive literature suggesting that Behavioral Inhibition is a predictor of SA as well as a vulnerability factor for SAD, Anxiety Sensitivity is also an important contributor in the development of SA in adolescence and Experiential Avoidance acts as a mediator (Berman et al., 2010; Fox and Kalin, 2014; Panayiotou et al., 2014; Pérez-Edgar and Guyer, 2014; Papachristou et al., 2018). Previous studies approach SA as a unitary construct mostly and do not cover relationships of specific dimensions/SA factors with temperament, which may offer us valuable information in personalizing interventions. For example, in our results, the relationship between “fear of Performance” and temperament shows us that anxiety sensitivity and experiential avoidance are more related with it than BI, which could have implications for early interventions. Similarly, if we assume that there are individual profiles distinct from one another and based on the number and type of fears and severity of symptoms, one would expect a different approach in each case. This requires an understanding of the relationships between temperament and specific SA dimensions.

The current results suggest that SPAI-23, and specifically the Greek-translation, can also be used for large scale screenings in the general population and it has the capacity to detect common dimensions of social fears. Furthermore, social fears in Greek-Cypriot adolescents are similar to those in other countries and they have similar links with temperamental characteristics. This, supports the idea that SA is a construct cross-culturally invariant along with other characteristics in the general population at that age-group. Further research is required to investigate potential groups of community adolescents exhibiting distinct fears that may cluster together, forming profiles of SA, and comparing them with respect to temperamental characteristics.

A limitation of this study is that an abbreviated version of a questionnaire was used to investigate dimensions of SA and it is unlikely that a reduced number of items can reflect all dimensions reported in the literature where longer versions of questionnaires are used. Moreover, SPAI is a widely used instrument and many of the findings supporting the existence of certain dimensions may stem from studies that used the same instrument – which may not always emerge when using the abbreviated form of the questionnaire. Subsequently, this indicates a smaller pool of empirical findings with which the present results can be compared with. Further research is required, with additional samples from around the world, to conclude on the most replicable subdimensions of social fears. Also, large epidemiological studies will need to examine the prevalence and overlap of these fears in youth and adults, of various ages, and different levels of SA severity. Moreover, the correlation analyses do not offer novel information on the relationships between SA factors and temperament and they mostly replicate previous findings; their main contribution is that temperamental characteristics may contribute in a unique way to different social fears. Another shortcoming of this study is that it used a community sample of adolescents. The results do not represent adolescents with clinical levels of SA, even though our sample was randomly selected, and therefore could potentially include subjects with clinical levels of SA. Results should be replicated using large subclinical and clinical samples. Yet, considering a dimensional approach that places individuals along a continuum depending on the severity of the symptoms, such as the RDoC (Insel et al., 2010), our results should be considered representative of adolescents in the “normal” side of that continuum. There are additional studies assessing the dimensionality of SAD and approach it as non-categorical (Crome et al., 2010; Ruscio, 2010; Fuentes-Rodriguez et al., 2018; Zsido et al., 2021), which poses important implications for future research, assessment and intervention design.

Examining dimensions of SA and SAD and identifying clusters of social fears intends to improve detection and intervention methods and, thus, has primarily clinical implications (Vriends et al., 2007; Kodal et al., 2017). Specifically, the presence of more social fears (i.e., a more generalized disorder) indicate increased severity of SAD, more comorbid disorders, increased dysfunctional attitudes, poorer mental health and more functional impairment overall (Stein et al., 2000; Vriends et al., 2007). Moreover, it has been shown that people with more severe SA symptoms may experience also a wider array of inter-correlated symptoms (Panayiotou et al., 2017). Further research on the prevalence and overlap of the identified dimensions of social fears will allow for an assessment whether the current classification adequately captures disorder presentations, or whether further subtyping is required. Previous findings especially those derived from studies using SPAI-C, suggest the existence of at least three dimensions that relate to “performance,” “interaction” and “being observed” (Bögels et al., 2010; Kodal et al., 2017). Our study confirms these findings or the presence of at least these categories of social fears.

In conclusion, a degree of uncertainty in the definition of subtypes is expected given the heterogeneity of SA (Kopala-Sibley et al., 2014; Binelli et al., 2015; Kodal et al., 2017). Not only are there too few studies exploring this topic in youth to draw firm conclusions, but these are also characterized by methodological differences in terms of population characteristics and assessment of SA. The resulting divergence in findings hinders interpretability and utilization of the results in clinical/ therapeutic settings. Nonetheless, having a broader understanding of SA manifestations has the potential to improve the clinical/therapeutic utility of the current diagnostic tools and overcome limitations of the categorical approaches, while being consistent with dimensional approaches in psychopathology (Hyett and McEvoy, 2018), such as the Research Domain Criteria (RDoC) (Insel et al., 2010).

## Data availability statement

The raw data supporting the conclusions of this article will be made available by the authors, without undue reservation.

## Ethics statement

The studies involving human participants were reviewed and approved by Cyprus National Bioethics Committee. Written informed consent to participate in this study was provided by the participants’ legal guardian/next of kin.

## Author contributions

GP conceptualized and designed the study. MT and KN conducted the research. MA analyzed the data and wrote the manuscript. MT and GP revised the manuscript. All authors contributed to the article and approved the submitted version.

## Funding

This study was funded by the Neo-PRISM-C project (European Union Horizon 2020 Program, H2020-MSCA-ITN2018) under the Marie Skłodowska-Curie Innovative Training Network (Grant Agreement No. 813546).

## Conflict of interest

The authors declare that the research was conducted in the absence of any commercial or financial relationships that could be construed as a potential conflict of interest.

## Publisher’s note

All claims expressed in this article are solely those of the authors and do not necessarily represent those of their affiliated organizations, or those of the publisher, the editors and the reviewers. Any product that may be evaluated in this article, or claim that may be made by its manufacturer, is not guaranteed or endorsed by the publisher.
